# Incident infection risks depending on oral antidiabetic exposure in insulin-treated type 2 diabetes patients

**DOI:** 10.1038/s41598-023-45793-x

**Published:** 2023-10-27

**Authors:** Sanghwa Park, Jiseon Jeong, Yunna Woo, Yeo Jin Choi, Sooyoung Shin

**Affiliations:** 1 College of Pharmacy, Ajou University, Suwon, Republic of Korea; 2 Department of Pharmacy, College of Pharmacy, Kyung Hee University, Seoul, Republic of Korea; 3 Research Institute of Pharmaceutical Science and Technology (RIPST), Ajou University, Suwon, Republic of Korea

**Keywords:** Diseases, Endocrinology, Health care

## Abstract

Dipeptidyl peptidase-4 inhibitors (DPP4is) and sodium glucose cotransporter-2 inhibitors (SGLT2is) have been speculated to have a potential to increase infection risks in type 2 diabetes mellitus (T2DM) patients. We performed a cohort study using the Korean health insurance data to investigate infection risks with each drug class relative to metformin in insulin-treated T2DM patients. After propensity score matching, we included 1,498 and 749 patients in DPP4i + insulin vs metformin + insulin and 300 and 549 patients in SGLT2i + insulin vs metformin + insulin, respectively. In stratified analyses per patient factor, none of the odds ratios (ORs) were associated with a statistical significance across respiratory, genital, and urinary tract infections (UTIs), except that of the male stratum for respiratory infections (OR 0.77, p = 0.04). With regard to SGLT2is, a higher risk of genital infections was analyzed with their use than with metformin therapy (OR 1.76, p = 0.03). In stratified analyses, the OR for genital infections remained significant in the baseline cardiovascular disease stratum (OR 2.29, p = 0.01). No increased UTI risk was detected with SGLT2is compared against metformin. In this study on insulin-receiving T2DM patients, DPP4is were not associated with increased infection risks, whereas SGLT2is led to a higher risk for genital infections, but not for UTIs, relative to metformin.

## Introduction

The prevalence of type 2 diabetes mellitus (T2DM) is increasing worldwide, and more patients are expected to receive glucose-lowering therapy^1^. Glycemic control in T2DM patients is of paramount importance as the disease is progressive in nature and poorly controlled T2DM can contribute to the development of not only severe cardiovascular (CV) and renal complications, but also cancer and dementia^2^. In addition to dietary and lifestyle modifications, early-stage disease patients are likely to be initiated on an oral antidiabetic, especially metformin, the first-line T2DM therapy, but in patients with progressed disease a combination glucose-lowering regimen composed of oral antidiabetic and parenteral insulin therapy is prevalently used^3^. These patient populations are susceptible to adverse health outcomes due to life-long exposure to hypoglycemic therapy and the attributes of T2DM itself that typically progresses to various metabolic dysfunctions^4^. Recently, it has been speculated in a series of studies that certain glucose-lowering agents, particularly dipeptidyl peptidase-4 (DPP-4) inhibitors and sodium glucose cotransporter-2 (SGLT-2) inhibitors, might be associated with increased risk of infectious diseases^5–7^. Given that the prescription volume of the two antidiabetic classes is steadily on the rise^8^, a potential link between their use and infection risks, albeit still controversial, may well leave healthcare professionals worldwide uncertain about optimal glycemic control regimens for T2DM patients who are already predisposed to infections due to diabetes-associated impaired immune responses^9–11^.

DPP-4 inhibitors are guidelines-recommended second- or third-line antidiabetic therapy with a decreased risk of hypoglycemia, cardiovascular disease and weight gain^12–15^. They are not only a common add-on therapy to the first-line metformin in antidiabetic treatment escalation plans but also frequently combined with insulin in later-stage T2DM patients^16,17^. Inhibition of DPP-4’s enzymatic activity potentiates glucagon-like peptide-1 (GLP-1) signaling and facilitates insulin secretion, and thereby controls glucose levels in diabetic patients^18^. In addition to glucose homeostasis, DPP-4 may also play a role in immune modulation as it has other substrates than incretin hormones, such as cytokines and chemokines that facilitate innate immune and inflammation pathways^19^. The underlying mechanisms have not been fully elucidated, but speculation has arisen over DPP-4 inhibitors’ potential effects on diabetic patients’ immune responses, which could debilitate patients’ immune response to respiratory tract infections, sepsis and other severe infections^20,21^.

Another controversial class of antidiabetics with respect to infection risks is the SGLT-2 inhibitor which has been associated with a higher risk of genitourinary tract infections than other antidiabetics due to its hypoglycemic mechanism: SGLT-2 inhibitors downregulate glucose reabsorption by blocking SGLT-2 protein in renal tubules and promote the urinary excretion of glucose^22,23^. In 2015, the US Food and Drug Administration (FDA) released a safety warning that SGLT-2 inhibitors may make diabetic patients susceptible to urinary tract infections (UTIs)^24^. Previous studies demonstrated that SGLT-2 inhibitor use led to elevated genital infection risks^25^; however, mixed results have been reported thus far regarding its potential to increase UTI risks^26–32^. With the first agent being approved in 2013, the real-world safety profile of SGLT-2 inhibitors has not been fully investigated. In light of its relatively short track records on the market compared to other antidiabetic agents, more pharmacovigilance studies are required to verify the potential association between these agents and urogenital infections.

Uncontrolled diabetes is thought to contribute to the immune system dysfunctions, resulting in diabetic patients being more likely to experience infectious diseases and adverse outcomes from them compared to non-diabetic adults^9–11^. It has been suggested that diabetes is linked to an increased pneumonia risk and higher mortality after pneumonia relative to non-diabetic patients^33–37^. Indeed, diabetes has been assessed as a risk factor for severe pneumonia and death in those affected by 2019 coronavirus diseases (COVID-19)^38–41^, and it remains uncertain whether certain antidiabetic agents with potential immune-modulating effects are safe to use in diabetic patient populations. Considering their life-long dependency on hypoglycemic therapy to prevent CV and metabolic complications due to T2DM, there is a strong need to investigate potential infectious disease risks linked to major antidiabetic drug classes, such as DPP-4 inhibitors and SGLT-2 inhibitors. Later-stage T2DM patients who are at higher risks for adverse health outcomes than early-stage patients were often not well represented in clinical studies. Hence, in this real-world data (RWD)-based cohort study, we aim to evaluate differential risks of infections focusing on respiratory and urogenital systems associated with DPP-4 inhibitors and SGLT-2 inhibitors as compared to metformin therapy in insulin-treated diabetic patients with progressed T2DM states.

## Results

### Characteristics of study patients

**Figure 1:**
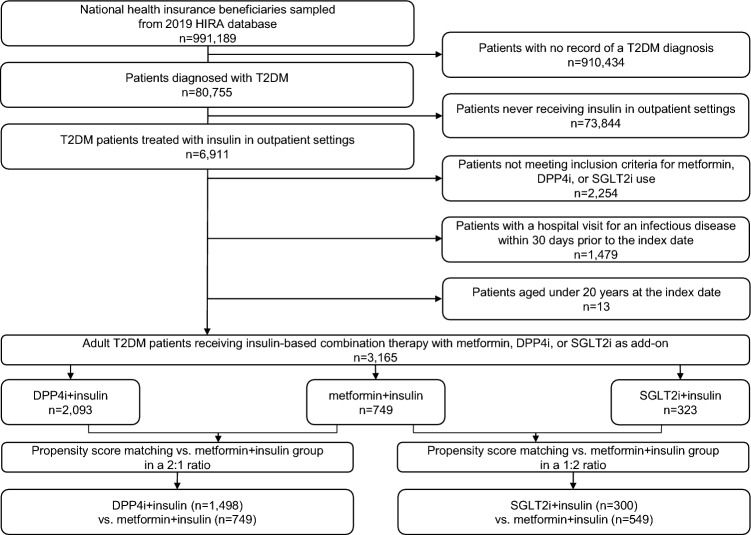
Flow diagram of the process of identifying and selecting study patients. *HIRA* health insurance review and assessment service, *T2DM* type 2 diabetes mellitus, *DPP4i* dipeptidyl peptidase-4 inhibitor, *SGLT2i* sodium glucose cotransporter-2 inhibitor.

Of the entire national patients registered in the Korean Health Insurance Review and Assessment Service (HIRA) database in 2019, a total of 991,189 patients were included in the initial patient sample (about 2% of the national health insurance beneficiaries in South Korea). There were 80,755 adult patients with a T2DM diagnosis in the initial cohort. Of those, the number of T2DM patients treated with insulin as outpatients was 6911. Of those, 749, 2093, and 323 patients concomitantly received metformin, DPP-4 inhibitor, SGLT-2 inhibitor therapy as oral antidiabetic comedication, respectively, in addition to insulin therapy. Baseline characteristics of the initial cohort are summarized in Table S1. After propensity score (PS) matching, 1498 and 749 patients were identified in the DPP-4 inhibitor + insulin versus metformin + insulin (reference) comparison pair while 300 and 549 patients were identified in the SGLT-2 inhibitor + insulin versus metformin + insulin (reference) comparison pair, respectively. The baseline characteristics of the PS-matched groups are also described in Table 1. The selection process of patients into each analytic cohort is depicted in Fig. 1. After PS matching, there were no significant differences between groups with regard to patient age, sex, Charlson comorbidity index (CCI), and comorbidities. However, between-group distributions in some of the oral antidiabetic comedication patterns were not completely balanced with PS matching, for which our risk analyses Underwent further statistical adjustment.

**Table 1:** Baseline characteristics of PS-matched progressed T2DM patients receiving DPP4i + insulin and SGLT2i + insulin vs metformin + insulin combination therapy each.

	DPP4i + insulin (n = 1498)	Metformin + insulin (n = 749)	SGLT2i + insulin (n = 300)	Metformin + insulin (n = 549)
Age group				
20 to < 65 years	553 (36.9)	268 (35.8)	161 (53.7)	268 (48.8)
65 to < 80 years	663(44.3)	345 (46.1)	122 (40.7)	247 (45.0)
≥ 80 years	282 (18.8)	136 (18.2)	17 (5.7)	34 (6.2)
Sex				
Male	832 (55.5)	427 (57.0)	155 (51.7)	307 (55.9)
Female	666 (44.5)	322 (43.0)	145 (48.3)	242 (44.1)
CCI	2.6 ± 2.2	2.4 ± 2.1	2.5 ± 2.0	2.3 ± 1.96
≤ 1	550 (36.7)	288 (38.5)	109 (36.3)	217 (39.5)
2	261(17.4)	137 (18.3)	55 (18.3)	108 (19.7)
≥ 3	687 (45.9)	324 (43.3)	136 (45.3)	224 (40.8)
CVD	1,155 (77.1)	564 (75.3)	226 (75.3)	395 (71.9)
DM with complications	958 (64.0)	447 (59.7)	194 (64.7)	324 (59.0)
Chronic lower respiratory disease	498 (33.2)	234 (31.2)	102 (34.0)	153 (27.9)
Renal disease	106 (7.1)	53 (7.1)	17 (5.7)	40 (7.3)
Pregnancy	9/666 (1.4)	5/322 (1.6)	1/145 (0.7)	5/242 (2.1)
Cancer	129 (8.6)	67 (8.9)	24 (8.0)	42 (7.7)
Comedication				
SU	470 (31.4)	296 (39.5)	56 (18.7)	209 (38.1)
TZD	140 (9.3)	63 (8.4)	7 (2.3)	50 (9.1)
Meglitinide	10 (0.7)	14 (1.9)	4 (1.3)	7 (1.3)
Alpha-glucosidase inhibitor	23 (1.5)	19 (2.5)	3 (1.0)	11 (2.0)
GLP-1 analog	20 (1.3)	52 (6.9)	8 (2.7)	43 (7.8)
Immunosuppressant	29 (1.9)	17 (2.3)	4 (1.3)	12 (2.2)
Glucocorticoid	335 (22.4)	132 (17.6)	62 (20.7)	91 (16.6)

*PS* propensity score, *T2DM* type 2 diabetes mellitus, *DPP4i* dipeptidyl peptidase-4 inhibitor, *SGLT2i* sodium glucose cotransporter-2 inhibitor, *CCI* Charlson comorbidity index, *CVD* cardiovascular disease, *DM* diabetes mellitus, *SU* sulfonylurea, *TZD* thiazolidinedione, *GLP-1* glucagon-like peptide-1.

### Study outcomes: DPP-4 inhibitor + insulin versus metformin + insulin

**Figure 2:**
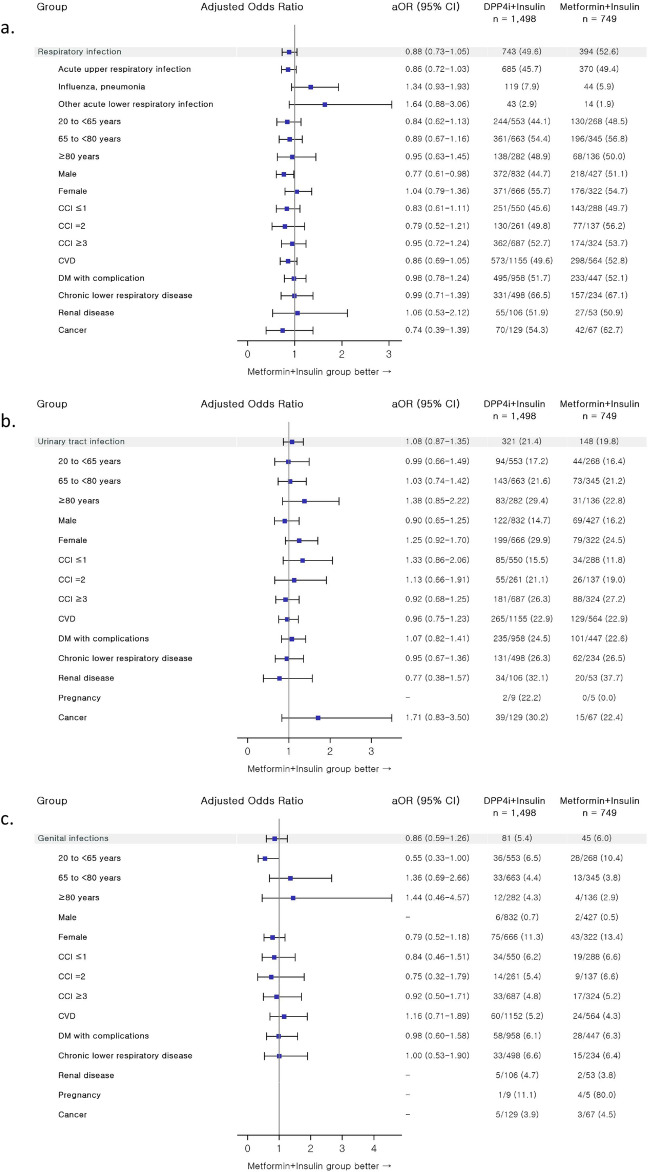
Forest plot for infection risks of DPP4i + insulin vs metformin + insulin combination therapy in progressed DM patients: (**a**) respiratory infection; (**b**) urinary tract infection; (**c**) genital infection. Risk analyses were adjusted for comedication patterns of sulfonylurea, meglitinide, glucagon-like peptide-1 analog, and glucocorticoid. *aOR* adjusted odds ratio, *CI* confidence interval, *DPP4i* dipeptidyl peptidase-4 inhibitor, *CCI* Charlson comorbidity index, *CVD* cardiovascular disease, *DM* diabetes mellitus.

The incidence and risk of new-onset infections in DPP-4 inhibitor + insulin users versus metformin + insulin users were analyzed, and the results are summarized in Fig. 2 and Table S2 in the supplementary material. Infection risk analyses were categorized into three outcome sites: respiratory infections, UTIs, and genital infections. The overall risk of respiratory infection (a composite endpoint event of acute upper respiratory infection, influenza and pneumonia, and other acute lower respiratory infection) was comparable between treatment groups, except for the male stratum where the respiratory infection risk was assessed lower with the concomitant use of DPP-4 inhibitors as compared to metformin use in insulin-treated T2DM patients, with an adjusted odds ratio (OR) and 95% confidence interval (CI) of 0.77 and 0.61–0.98 (Table S2). With regard to each component of respiratory infections as well as each stratum per patient factor (age, gender, and comorbidity), no significant risk of respiratory infections was assessed among DPP-4 inhibitor + insulin users as compared to metformin + insulin users. Similar results were found with UTI and genital infection risks: these infection risks were comparable between comparison groups in each outcome category as a whole and across all strata per patient factor in subsequent stratified risk analyses.

### Study outcomes: SGLT-2 inhibitor + insulin versus metformin + insulin

**Figure 3:**
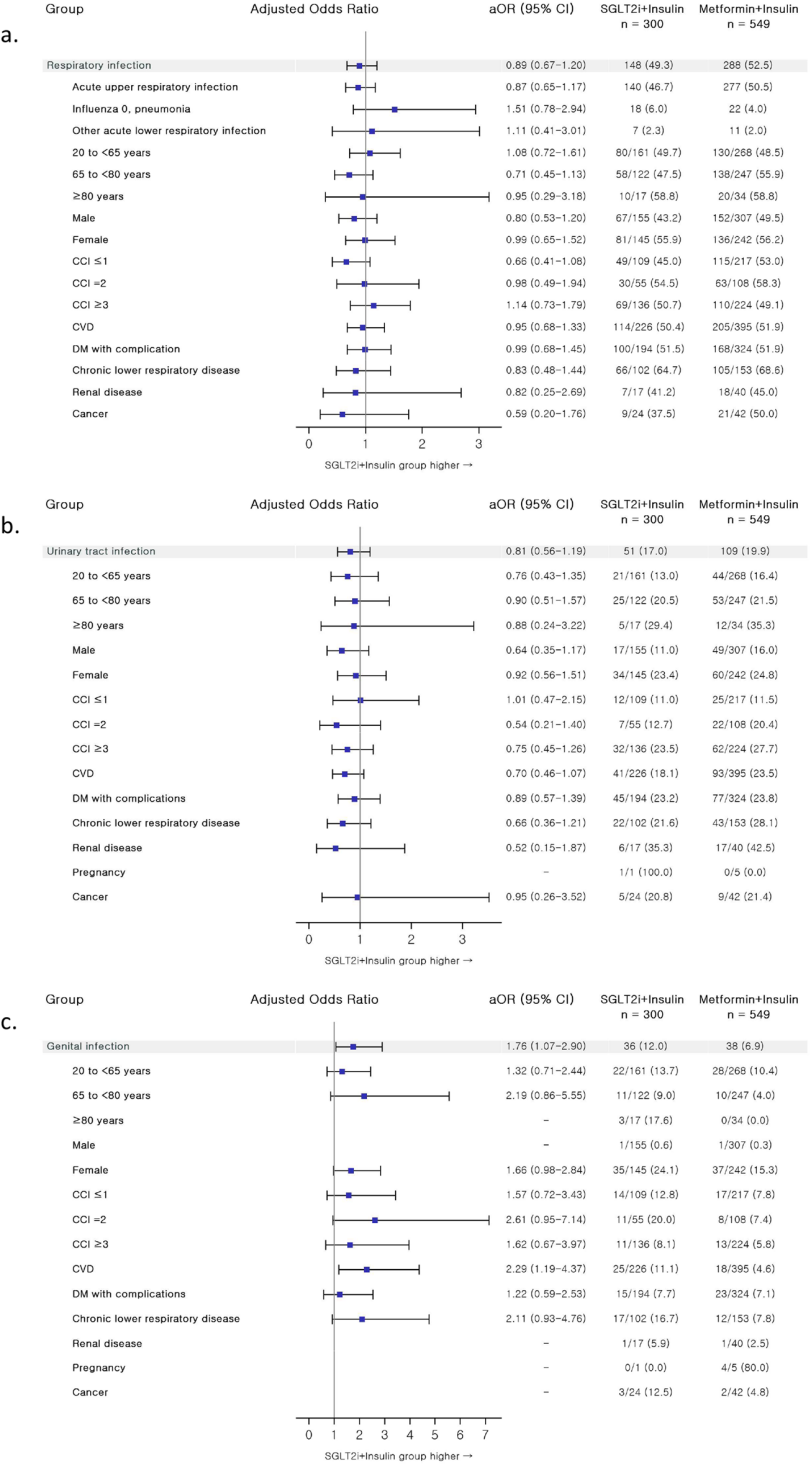
Forest plot for infection risks of SGLT2i + insulin vs metformin + insulin combination therapy in progressed DM patients: (**a**) respiratory infection; (**b**) urinary tract infection; (**c**) genital infection. Risk analyses were adjusted for comedication patterns of sulfonylurea, thiazolidinedione, and glucagon-like peptide-1 analog. *aOR* adjusted odds ratio, *CI* confidence interval, *SGLT2i* sodium glucose cotransporter-2 inhibitor, *CCI* Charlson comorbidity index, *CVD* cardiovascular disease, *DM* diabetes mellitus.

The incidence and risk of new-onset infections in SGLT-2 inhibitor + insulin users versus metformin + insulin users were analyzed, and the results are summarized in Fig. 3 and Table S3. The risk of genital infections was elevated with SGLT-2 inhibitors as compared to metformin comedication in insulin-treated T2DM patients with an OR (95% CI) of 1.76 (1.07–2.90) (Table S3). Following stratification per patient factor, insulin-treated T2DM patients with underlying cardiovascular diseases (CVDs) were at a greater risk of developing genital infections when exposed to SGLT-2 inhibitors compared against metformin-receiving patients, with an adjusted OR (92% CI) was 2.29 (1.19–4.37). Additionally, in crude OR analyses, SGLT-2 inhibitor exposure was associated with a higher genital infection risk in the following strata: females, CCI of 2, and baseline comorbidity of chronic lower respiratory disease, with crude ORs of 1.76 (p = 0.03), 3.13 (p = 0.02) and 2.35 (p = 0.03), respectively. However, in subsequent adjusted OR analyses, none of these strata showed a statistically significant association between genital infection risks and combined use of SGLT-2 inhibitors compared against metformin in insulin-treated T2DM patients. The incidence of genital infections was higher with SGLT-2 inhibitor + insulin therapy than with metformin + insulin combination in the following strata: ≥ 80 years of age, male, and comorbid conditions of renal disease and cancer at baseline, but risk analyses were not performed due to the scarcity of endpoint events as well as the limited sample size of these strata.

## Discussion

In this retrospective analysis of the real-world health data, we performed a retrospective cohort study to investigate differential infection risks of oral antidiabetics in insulin-treated T2DM patients by designing two comparison pairs: (1) DPP-4 inhibitor + insulin combination versus metformin + insulin combination; (2) SGLT-2 inhibitor + insulin combination versus metformin + insulin combination. We found that the risk of respiratory infections was lower when DPP-4 inhibitor therapy was used concomitantly with insulin, relative to the metformin + insulin combination regimen in male T2DM patients (adjusted OR 0.77; 95% CI 0.61–0.98, p = 0.04). However, in our stratified analyses per patient factor (sex, age, CCI, and comorbidity), none of the ORs across all three outcome sites (respiratory, urinary tract, and genital systems) except that of the male stratum for respiratory infections were associated with a statistical significance when compared against the corresponding metformin + insulin group, respectively. With regard to SGLT-2 inhibitors, a substantially higher risk of genital infections was analyzed with their use than in those using metformin in insulin-receiving T2DM patients (adjusted OR 1.76; 95% CI 1.07–2.90, p = 0.03). When the analysis was repeated using stratified methods, the OR for genital infections remained significant post statistical adjustments only in those patients having CVD comorbid conditions at study entry (adjusted OR 2.29; 95% CI 1.19–4.37, p = 0.01). The CVD stratum patients likely had more hospital visit episodes than those without CVDs as baseline comorbidity, which might have affected the rates of patients being exposed to infectious pathogens.

Diabetes itself is a well-known risk factor for infections as high blood glucose, compromised immune systems, and comorbid conditions can predispose patients to infectious disorders^9–11^. Safety signals have been detected thus far primarily with two major oral antidiabetic classes, DPP-4 inhibitors and SGLT-2 inhibitors, in terms of their potential to increase the risk of respiratory and urogenital infections, respectively^5–7,20–23^. To the best of our knowledge, this is the first study that defined the study patients as only those with advanced T2DM already on insulin treatment and investigated the effects of DPP-4 inhibitors and SGLT-2 inhibitors on infectious disease risks as compared to metformin effects in these patient populations.

It has been speculated that DPP-4 inhibitors have a potential immunomodulating effect, but the mechanism behind it has yet to be elucidated^19–21^. In the present study, we compared the risk of respiratory infection (a composite endpoint event of acute upper respiratory infections, influenza, pneumonia and other acute lower respiratory infections) associated with DPP-4 inhibitor + insulin versus metformin + insulin combination therapy in progressed T2DM patients. As opposed to previous speculations over DPP-4 inhibitors’ potential link to pneumonia risks, the current study findings indicate that DPP-4 inhibitors pose no detrimental effects on respiratory tract infections, but rather exert protective effects especially in male patients with progressed T2DM already on insulin therapy. These results are consistent with those of two prior studies based on the UK primary care database and the Italian administrative database each, where an approximately 30% reduction in pneumonia risk with DPP-4 inhibitor use relative to other second-line oral glucose-lowering therapy has been reported^5,42^. Another study using a Spanish general practice research database and a meta-analysis of randomized controlled trials (RCTs), on the other hand, showed no protective effects against pneumonia or respiratory infections with DPP-4 inhibitor use compared against active comparator drugs^43,44^. Interestingly, in a previous study on Japanese patients, DPP-4 inhibitors also showed protective effects against UTIs relative to metformin with the intention-to-treat hazard ratio (HR) of 0.85 (0.71–0.94) and the per-protocol HR of 0.83 (0.71–0.95)^6^. In the present study, however, no beneficial effects with respect to UTIs were detected, which was also confirmed in a prior meta-analysis of RCTs^44^. Of note is the distinct difference in patient characteristics between prior studies and the current study: the present study incorporated only those patients with more advanced T2DM already dependent on insulin therapy whereas previous studies mostly enrolled T2DM patients irrespective of their disease progression status or insulin use history.

When pooling the estimated effects from previous studies and ours, the overall risk of respiratory infections linked to DPP-4 inhibitors is still inconclusive^5,42,43^. In the current study, a statistically significant OR was observed only in the male stratum, with the upper limit of the 95% CI was just below 1.0. Further studies are required to confirm whether DPP-4 inhibitors would exert clinically significant protective effects against respiratory infections. Nevertheless, several clinical studies and meta-analyses thus far all suggest that DPP-4 inhibitors are not associated with elevated risk of infectious diseases as compared to metformin^5,42–46^. However, caution is advised when interpreting these results as there exist disparities in study designs and characteristics of included patients across the aforementioned studies.

SGLT-2 inhibitors are a relatively new oral antidiabetic class, and in most countries SGLT-2 inhibitors are used as a second-line option but in Japan these agents are one of the first-line therapies for T2DM^12,47^. Safety concerns have been expressed about their UTI risks as these agents lower serum glucose levels by inducing glucosuria, which can adversely promote bacterial growth in the urinary system^22,23^. Following the 2015 warning by the FDA about severe UTI risks, their labels were updated to include UTIs as potential adverse events^24^. A meta-analysis of RCTs showed that SGLT-2 inhibitors were associated with UTI risks compared to placebo^48^, but other meta-analyses and pharmacoepidemiological studies found no link between SGLT-2 inhibitors and UTI risks^26–31^. A 2021 study on Japanese early-stage T2DM patients analyzed the hazard of UTIs associated with SGLT-2 inhibitors relative to metformin using both the intention-to-treat and per-protocol methods to account for potential effects from treatment changes (additions and discontinuations) and found that SGLT-2 inhibitors did not increase the UTI risks regardless of treatment changes during the follow-up^6^. In line with these findings, our study results also showed no detrimental effects on UTI risks exerted by SGLT-2 inhibitors relative to metformin in insulin-treated T2DM patients.

Meanwhile, the SGLT-2 inhibitor-induced glucosuria can also contribute to the development of genital infections by facilitating commensal organisms’ growth in urine^22,23^. In 2018, the FDA warned that serious genital infections may occur with SGLT-2 inhibitor use^25^. Our study findings suggested that insulin-receiving T2DM patients were at a higher risk of genital infections when exposed to SGLT-2 inhibitor therapy relative to metformin as comedication (adjusted OR 1.76, p = 0.03). Prior retrospective cohort studies in Australia and in the US, a systematic review in China and other studies also showed that SGLT-2 inhibitor users experienced higher rates of genital infections than with other oral antidiabetics^28,32,49^. However, a signal of UTI risks was not detected with SGLT-2 inhibitors in these studies^28,32,49^. The current study findings were comparable to these study results in that SGLT-2 inhibitors were associated with a higher genital infection risk but not with a UTI risk relative to metformin in insulin-treated T2DM patients.

Over the last decade, DPP-4 inhibitors have shown a steady uptake in prescription volume, and due to their preferable safety profiles they have been preferably used in T2DM patients with chronic kidney diseases and in those intolerant to adverse effects from other oral antidiabetics^8,50^. With respect to SGLT-2 inhibitors, studies demonstrated their beneficial effects on cardiac events, strokes and mortality outcomes in T2DM patients with CV comorbid conditions, along with a lower risk of hypoglycemia than other oral antidiabetics^51–54^. The current study findings offer further assurance that DPP-4 inhibitors are safe to be used together with insulin in progressed T2DM patients in terms of infectious disease risks, whereas the link between SGLT-2 inhibitors and genital infection risks was also confirmed in our study.

### Limitations

This study has several limitations. First, endpoint events and patient comorbid conditions were tracked and identified based on the International Classification of Disease 10th Revision (ICD-10) diagnosis codes; therefore, inaccurate or missing documentation of diagnostic codes in the HIRA data may lead to under- or over-estimation of the distribution of comorbid conditions between comparison groups as well as the frequency of study outcome events. Second, initial patient data of a single-year cohort were extracted based on stratified randomized sampling methods from the HIRA database. Further RWD studies with a larger sample size and an extended follow-up period is required to verify the current study findings and to evaluate long-term safety of DPP-4 inhibitors and SGLT-2 inhibitors in terms of new-onset infections when combined with insulin in later-stage T2DM patients. Lastly, even though PS-matching and adjusted multiple logistic regression methods were employed in this study, residual confounding effects can still exist that are associated with unidentified covariates, such as comedication patterns with SUs, TZDs, meglitinides, etc. However, we assume the aforementioned confounding effects would be minimal as no significant infection risk has been associated thus far with these oral antidiabetics. Also, the prescription volume of these agents is on the steady decrease over time or remains negligible as newer and more effective oral antidiabetics with preferable side effect profiles, such as DPP-4 inhibitors and SGLT-2 inhibitors, became available for glycemic control.

## Conclusions

In this study, we investigated a potential risk of infectious diseases with the use of two major oral antidiabetics, DPP-4 inhibitors and SGLT-2 inhibitors. Our study results demonstrated that in progressed T2DM patients already on insulin treatment in outpatient settings, neither detrimental nor beneficial effects were exerted by DPP-4 inhibitor comedication in terms of infection risks, whereas those exposed to SGLT-2 inhibitors were at higher risk of genital infections when compared against those using metformin as comedication.

## Materials and methods

### Study population

A retrospective cohort study in later-stage T2DM patients treated with insulin, in combination with metformin, DPP-4 inhibitors or SGLT-2 inhibitors, was performed using the HIRA National Patients Sample-2019 (NPS-2019) data. The HIRA database contains National Health Insurance (NHI) claims administrative data related to health-care services conferred to the entire beneficiaries in South Korea, including patient demographics, ICD-10-based diagnosis codes, inpatient and outpatient medical utilization data, and comprehensive prescription data. The initial sampled cohort of 991,189 patients or 2% of the entire national populations in 2019 had been selected based on stratified randomized sampling methods to ensure national representativeness of the NPS data. Those patients, aged 20 years or above, with hospital encounters between January 1, 2019 and December 31, 2019, who were treated with metformin, DPP-4 inhibitor or SGLT-2 inhibitor therapy in addition to insulin therapy under the diagnosis of T2DM (identified per ICD-10 code, E11) were first screened for the eligibility for the study cohort. Antidiabetic therapy per drug class should last for more than 30 consecutive days to be considered as relevant therapeutic management. We excluded those patients who received insulin treatment during hospitalization only or in inpatient settings only and those treated with metformin and either of the two study antidiabetic drugs concomitantly as combination therapy. In addition, those patients who had been on both DPP-4 inhibitor and SGLT-2 inhibitor therapies for an equivalent duration (the number of prescription days not differing by over 50% of each other’s) were excluded from the study, resulting in two mutually-exclusive comparison pairs. Other exclusion criteria included those with a history of infectious diseases within 30 days prior to study entry. The study protocol was approved by the Institutional Review Board (IRB) of Ajou University (202201-HB-EX-003). All research was performed in accordance with relevant guidelines and regulations. Due to the retrospective nature of study, the need of informed consent was waived by the IRB of Ajou University. No further ethics approval was required as the authors are authorized by the HIRA to use the de-identified patient data for research purposes.

### Study medications and variables

Insulin-receiving T2DM patients concomitantly treated with metformin, DPP-4 inhibitors, or SGLT-2 inhibitors, with the therapy duration of more than 30 days each, were assigned to insulin + DPP-4 inhibitor users or insulin + SGLT-2 inhibitor users versus insulin + metformin users (reference) according to oral antidiabetic combination history. To include T2DM patients with progressed or later-stage disease, only those already dependent on insulin therapy in outpatient settings were eligible for study inclusion. Use of other oral antidiabetic therapies were permitted in each comparison group. Prespecified variables included patient demographics (age and sex), and CCI, comorbid conditions (such as diabetes mellitus with complications, chronic lower respiratory disease, and renal disease), comedication patterns (sulfonylurea, thiazolidinedione, meglitinide, α-glucosidase inhibitor, GLP-1 analog, and glucocorticoid). Comedication patterns of corticosteroid were also identified in each patient in consideration of its prescription volume as well as its potential to suppress immunity, thereby increasing infection risks in patients. The index date was defined as the date when oral antidiabetic therapy (metformin, DPP-4 inhibitor, or SGLT-2 inhibitor) was initiated during the study period. The consecutive therapy was defined as when a study antidiabetic was re-prescribed within 1.5 times the days-supply added to the prior prescription date after its end date. Those patients who had been on both comparison drugs for an equivalent duration (the number of prescription days not differing by over 50% of each other’s) were excluded from the study, resulting in two mutually-exclusive comparison pairs.

### Study outcomes

The primary outcome was the incidence and risk of infectious diseases (respiratory, genital, and UTIs) in insulin-treated T2DM patients who also received DPP-4 inhibitors or SGLT-2 inhibitors as co-medication as compared to metformin (reference). Incident infections assessed in this study include respiratory infections ranging from acute upper respiratory infections, influenza and pneumonia to other acute lower respiratory infections, urinary tract infections, and genital organ infections. Some of the diagnosis codes for UTIs and genital infections are sex specific. UTIs range from cystitis, pyelonephritis, urethritis, inflammatory diseases of the prostate for males and urethral syndrome for females. Genital infections are defined as a composite endpoint event encompassing Candida infections, vulvitis, vaginitis, gonococcal infections, and inflammatory disease of the uterus for females, while candidal balanitis, balanoposthitis, orchitis and epididymitis were considered as the outcome events for males. The risk analysis per infection site was further stratified by sex, CCI groups, comorbidities, and fibrate use in order to account for differential effects of patient factors on infection risks. In consideration of time to steady state plasma concentrations and the half-life of antidiabetic agents, only those outcome events that patients experienced at least 7 days post the index date were assessed valid and incorporated into study analyses. The outcome date was determined as the earliest date a patient encountered a given outcome event. The follow-up period started on the index date and lasted until the earliest occurrence of any of the following: an outcome event, therapy discontinuation, death, or the end of study period (December 31, 2019).

### Statistical analysis

The incidence and risk of new-onset infections in each comparison pair were calculated with ORs and 95% CIs of endpoint events. We assumed that the OR which does not consider rates or timeline would be appropriate for our study analyses as this retrospective cohort study based on a single year national insurance data had a relatively short study period. A *p*-value of < 0.05 was assessed statistically significant. To minimize possible confounding effects due to between-group differences across baseline variables, PS matching was performed and repeated for each comparison pair. The caliper matching method was used at a ratio of 2:1 and 1:2 for the DPP-4 inhibitor + insulin vs metformin + insulin comparison pair and for the SGLT-2 inhibitor + insulin vs metformin + insulin comparison pair, respectively. A multivariable logistic regression model was utilized to estimate the PS for individual patients, which predicts the probability of patient exposure to DPP-4 inhibitor therapy or SGLT-2 inhibitor therapy versus metformin therapy (reference) among insulin-treated T2DM patients given prespecified baseline variables. The baseline variables incorporated in a multivariable logistic regression as covariates were age groups, sex, CCI groups, and comorbidities. Statistical analyses were carried out with SAS 9.4 Software (SAS Institute Inc., Cary, NC, USA).

### Supplementary Information

Supplementary Information.

## Data Availability

Data are available from the corresponding author upon reasonable request. Restrictions may apply to the availability of some of the data, which were used under permission from the HIRA.
