# Supplementary material for: Incident infection risks depending on oral antidiabetic exposure in insulin-treated type 2 diabetes patients
Source: Sci Rep. 2023 Oct 27;13:18462. doi: 10.1038/s41598-023-45793-x (PMC10611756; doi:10.1038/s41598-023-45793-x)
Supplement: Supplementary file 1 — Supplementary Information. [file 41598_2023_45793_MOESM1_ESM.docx]

**Supplementary Materials**

Table S1. Baseline characteristics of progressed T2DM patients receiving DPP4i+insulin and SGLT2i+insulin vs metformin+insulin combination therapy each (prior to PS-matching)

|  | DPP4i + Insulin  (n = 2,093) | Metformin + Insulin  (n = 749) | SGLT2i + Insulin  (n = 323) | Metformin + Insulin  (n = 749) |
| --- | --- | --- | --- | --- |
| Age group |  |  |  |  |
| 20 to <65 years | 684 (32.7) | 268 (35.8) | 184 (57.0) | 268 (35.8) |
| 65 to <80 years | 933 (44.6) | 345 (46.1) | 122 (37.8) | 345 (46.1) |
| ≥80 years | 476 (22. 7) | 136 (18.2) | 17 (5.3) | 136 (18.2) |
| Sex |  |  |  |  |
| Male | 1,163 (55.6) | 427 (57.0) | 155 (48.0) | 427 (57.0) |
| Female | 930 (44.4) | 322 (43.0) | 168 (52.0) | 322 (43.0) |
| CCI | 3.2±2.5 | 2.4±2.1 | 2.4±2.0 | 2.4±2.1 |
| ≤1 | 550 (26.3) | 288 (38.5) | 119 (36.8) | 288 (38.5) |
| 2 | 331 (15.8) | 137 (18.3) | 61 (18.9) | 137 (18.3) |
| ≥3 | 1,212 (57.9) | 324 (43.3) | 143 (44.3) | 324 (43.3) |
| CVD | 1,691 (80.8) | 564 (75.3) | 236 (73.1) | 564 (75.3) |
| DM with complications | 1,468 (70.1) | 447 (59.7) | 203 (62.8) | 447 (59.7) |
| Chronic lower respiratory disease | 833 (39.8) | 234 (31.2) | 109 (33.7) | 234 (31.2) |
| Renal disease | 469 (22.4) | 53 (7.1) | 18 (5.6) | 53 (7.1) |
| Pregnancy | 9/930 (1.0) | 5/322 (1.6) | 3/168 (1.8) | 5/322 (1.6) |
| Cancer | 301 (14.4) | 67 (8.9) | 27 (8.4) | 67 (9.0) |
| Comedication |  |  |  |  |
| SU | 708 (33.8) | 296 (39.5) | 61 (18.9) | 296 (39.5) |
| TZD | 212 (10.1) | 63 (8.4) | 8 (2.5) | 63 (8.4) |
| Meglitinide | 19 (0.9) | 14 (1.9) | 4 (1.2) | 14 (1.9) |
| Alpha-glucosidase inhibitor | 34 (1.6) | 19 (2.5) | 4 (1.2) | 19 (2.5) |
| GLP-1 analog | 25 (1.2) | 52 (6.9) | 9 (2.8) | 52 (6.9) |
| Immunosuppressant | 68 (3.2) | 17 (2.3) | 4 (1.2) | 17 (2.3) |
| Glucocorticoid | 515 (24.6) | 132 (17.6) | 65 (20.1) | 132 (17.6) |

Abbreviations: T2DM, type 2 diabetes mellitus; DPP4i, dipeptidyl peptidase-4 inhibitor; SGLT2i, sodium glucose cotransporter-2 inhibitor; PS, propensity score; CCI, Charlson comorbidity index; CVD, cardiovascular disease; DM, diabetes mellitus; SU, sulfonylurea; TZD, thiazolidinedione; GLP-1, glucagon-like peptide-1.

Table S2. PS-matched analysis of infection risks comparing DPP4i+insulin against metformin+insulin combination therapy in T2DM patients

| Outcomes | DPP4i + Insulin  n = 1,498 | Metformin + Insulin  n = 749 | OR (95% CI) | p-value | Adjusted OR (95% CI) | p-value |
| --- | --- | --- | --- | --- | --- | --- |
| Respiratory infection | 743 (49.6) | 394 (52.6) | 0.89 (0.74-1.06) | 0.18 | 0.88 (0.73-1.05) | 0.16 |
| Acute upper respiratory infection | 685 (45.7) | 370 (49.4) | 0.86 (0.72-1.03) | 0.10 | 0.86 (0.72-1.03) | 0.09 |
| Influenza, pneumonia | 119 (7.9) | 44 (5.9) | 1.38 (0.97-1.98) | 0.07 | 1.34 (0.93-1.93) | 0.12 |
| Other acute lower respiratory infection | 43 (2.9) | 14 (1.9) | 1.55 (0.84-2.85) | 0.15 | 1.64 (0.88-3.06) | 0.12 |
| 20 to <65 years | 244/553 (44.1) | 130/268 (48.5) | 0.84 (0.63-1.12) | 0.24 | 0.84 (0.62-1.13) | 0.25 |
| 65 to <80 years | 361/663 (54.4) | 196/345 (56.8) | 0.91 (0.70-1.18) | 0.47 | 0.89 (0.67-1.16) | 0.38 |
| ≥80 years | 138/282 (48.9) | 68/136 (50.0) | 0.96 (0.64-1.44) | 0.84 | 0.95 (0.63-1.45) | 0.83 |
| Male | 372/832 (44.7) | 218/427 (51.1) | **0.76 (0.61-0.98)** | **0.03** | **0.77 (0.61-0.98)** | **0.04** |
| Female | 371/666 (55.7) | 176/322 (54.7) | 1.04 (0.80-1.36) | 0.76 | 1.04 (0.79-1.36) | 0.81 |
| CCI ≤1 | 251/550 (45.6) | 143/288 (49.7) | 0.85 (0.64-1.13) | 0.27 | 0.83 (0.61-1.11) | 0.20 |
| CCI =2 | 130/261 (49.8) | 77/137 (56.2) | 0.77 (0.51-1.17) | 0.23 | 0.79 (0.52-1.21) | 0.28 |
| CCI ≥3 | 362/687 (52.7) | 174/324 (53.7) | 0.96 (0.74-1.25) | 0.76 | 0.95 (0.72-1.24) | 0.69 |
| CVD | 573/1155 (49.6) | 298/564 (52.8) | 0.88 (0.72-1.08) | 0.21 | 0.86 (0.69-1.05) | 0.14 |
| DM with complication | 495/958 (51.7) | 233/447 (52.1) | 0.98 (0.78-1.23) | 0.87 | 0.98 (0.78-1.24) | 0.89 |
| Chronic lower respiratory disease | 331/498 (66.5) | 157/234 (67.1) | 0.97 (0.70-1.35) | 0.87 | 0.99 (0.71-1.39) | 0.96 |
| Renal disease | 55/106 (51.9) | 27/53 (50.9) | 1.04 (0.54-2.01) | 0.91 | 1.06 (0.53-2.12) | 0.87 |
| Cancer | 70/129 (54.3) | 42/67 (62.7) | 0.71 (0.39-1.29) | 0.26 | 0.74 (0.39-1.39) | 0.35 |
| Urinary tract infection | 321 (21.4) | 148 (19.8) | 1.11 (0.89-1.38) | 0.36 | 1.08 (0.87-1.35) | 0.49 |
| 20 to <65 years | 94/553 (17.2) | 44/268 (16.4) | 1.06 (0.72-1.56) | 0.79 | 0.99 (0.66-1.49) | 0.96 |
| 65 to <80 years | 143/663 (21.6) | 73/345 (21.2) | 1.02 (0.75-1.41) | 0.88 | 1.03 (0.74-1.42) | 0.88 |
| ≥80 years | 83/282 (29.4) | 31/136 (22.8) | 1.41 (0.88-2.27) | 0.15 | 1.38 (0.85-2.22) | 0.19 |
| Male | 122/832 (14.7) | 69/427 (16.2) | 0.89 (0.65-1.23) | 0.48 | 0.90 (0.65-1.25) | 0.52 |
| Female | 199/666 (29.9) | 79/322 (24.5) | 1.31 (0.97-1.78) | 0.08 | 1.25 (0.92-1.70) | 0.16 |
| CCI ≤1 | 85/550 (15.5) | 34/288 (11.8) | 1.37 (0.89-2.09) | 0.15 | 1.33 (0.86-2.06) | 0.20 |
| CCI =2 | 55/261 (21.1) | 26/137 (19.0) | 1.14 (0.68-1.92) | 0.62 | 1.13 (0.66-1.91) | 0.66 |
| CCI ≥3 | 181/687 (26.3) | 88/324 (27.2) | 0.96 (0.71-1.29) | 0.78 | 0.92 (0.68-1.25) | 0.60 |
| CVD | 265/1155 (22.9) | 129/564 (22.9) | 1.00 (0.79-1.28) | 0.97 | 0.96 (0.75-1.23) | 0.74 |
| DM with complications | 235/958 (24.5) | 101/447 (22.6) | 1.11 (0.85-1.45) | 0.43 | 1.07 (0.82-1.41) | 0.62 |
| Chronic lower respiratory disease | 131/498 (26.3) | 62/234 (26.5) | 0.99 (0.70-1.41) | 0.96 | 0.95 (0.67-1.36) | 0.80 |
| Renal disease | 34/106 (32.1) | 20/53 (37.7) | 0.78 (0.39-1.55) | 0.48 | 0.77 (0.38-1.57) | 0.47 |
| Pregnancy | 2/9 (22.2) | 0/5 (0.0) | - | - | - | - |
| Cancer | 39/129 (30.2) | 15/67 (22.4) | 1.50 (0.76-2.98) | 0.24 | 1.71 (0.83-3.50) | 0.14 |
| Genital infections | 81 (5.4) | 45 (6.0) | 0.89 (0.61-1.30) | 0.56 | 0.86 (0.59-1.26) | 0.45 |
| 20 to <65 years | 36/553 (6.5) | 28/268 (10.4) | 0.60 (0.36-1.00) | 0.06 | 0.55 (0.33-1.00) | 0.05 |
| 65 to <80 years | 33/663 (4.4) | 13/345 (3.8) | 1.34 (0.69-2.58) | 0.38 | 1.36 (0.69-2.66) | 0.37 |
| ≥80 years | 12/282 (4.3) | 4/136 (2.9) | 1.47 (0.46-4.63) | 0.51 | 1.44 (0.46-4.57) | 0.53 |
| Male | 6/832 (0.7) | 2/427 (0.5) | - | - | - | - |
| Female | 75/666 (11.3) | 43/322 (13.4) | 0.82 (0.55-1.23) | 0.33 | 0.79 (0.52-1.18) | 0.25 |
| CCI ≤1 | 34/550 (6.2) | 19/288 (6.6) | 0.93 (0.52-1.67) | 0.81 | 0.84 (0.46-1.51) | 0.55 |
| CCI =2 | 14/261 (5.4) | 9/137 (6.6) | 0.81 (0.34-1.91) | 0.62 | 0.75 (0.32-1.79) | 0.52 |
| CCI ≥3 | 33/687 (4.8) | 17/324 (5.2) | 0.91 (0.50-1.66) | 0.76 | 0.92 (0.50-1.71) | 0.80 |
| CVD | 60/1152 (5.2) | 24/564 (4.3) | 1.24 (0.76-2.00) | 0.40 | 1.16 (0.71-1.89) | 0.56 |
| DM with complications | 58/958 (6.1) | 28/447 (6.3) | 0.96 (0.61-1.54) | 0.88 | 0.98 (0.60-1.58) | 0.92 |
| Chronic lower respiratory disease | 33/498 (6.6) | 15/234 (6.4) | 1.04 (0.55-1.95) | 0.91 | 1.00 (0.53-1.90) | 1.00 |
| Renal disease | 5/106 (4.7) | 2/53 (3.8) | - | - | - | - |
| Pregnancy | 1/9 (11.1) | 4/5 (80.0) | - | - | - | - |
| Cancer | 5/129 (3.9) | 3/67 (4.5) | - | - | - | - |

Notes: Risk analyses were adjusted for comedication patterns of sulfonylurea, meglitinide, and glucagon-like peptide-1 analog, and glucocorticoid. p-values were calculated with Chi-square test (Fisher’s exact test) for categorial variables. Statistically significant p-values are highlighted in bold.

Abbreviations: PS, propensity score; DPP4i, dipeptidyl peptidase-4 inhibitor; T2DM, type 2 diabetes mellitus; CCI, Charlson comorbidity index; CVD, cardiovascular disease; DM, diabetes mellitus.

Table S3. PS-matched analysis of infection risks comparing SGLT2i+insulin against metformin+insulin combination therapy in T2DM patients

| Outcomes | SGLT2i + Insulin  n = 300 | Metformin + Insulin  n = 549 | OR (95% CI) | p-value | Adjusted OR (95% CI) | p-value |
| --- | --- | --- | --- | --- | --- | --- |
| Respiratory infection | 148 (49.3) | 288 (52.5) | 0.88 (0.67-1.17) | 0.38 | 0.89 (0.67-1.20) | 0.45 |
| Acute upper respiratory infection | 140 (46.7) | 277 (50.5) | 0.86 (0.65-1.14) | 0.29 | 0.87 (0.65-1.17) | 0.36 |
| Influenza 0, pneumonia | 18 (6.0) | 22 (4.0) | 1.53 (0.81-2.90) | 0.19 | 1.51 (0.78-2.94) | 0.22 |
| Other acute lower respiratory infection | 7 (2.3) | 11 (2.0) | 1.17 (0.45-3.05) | 0.75 | 1.11 (0.41-3.01) | 0.83 |
| 20 to <65 years | 80/161 (49.7) | 130/268 (48.5) | 1.05 (0.71-1.55) | 0.81 | 1.08 (0.72-1.61) | 0.72 |
| 65 to <80 years | 58/122 (47.5) | 138/247 (55.9) | 0.72 (0.46-1.11) | 0.13 | 0.71 (0.45-1.13) | 0.15 |
| ≥80 years | 10/17 (58.8) | 20/34 (58.8) | 1.00 (0.31-3.26) | 1.00 | 0.95 (0.29-3.18) | 0.94 |
| Male | 67/155 (43.2) | 152/307 (49.5) | 0.78 (0.53-1.15) | 0.20 | 0.80 (0.53-1.20) | 0.28 |
| Female | 81/145 (55.9) | 136/242 (56.2) | 0.99 (0.65-1.49) | 0.95 | 0.99 (0.65-1.52) | 0.97 |
| CCI ≤1 | 49/109 (45.0) | 115/217 (53.0) | 0.72 (0.46-1.15) | 0.17 | 0.66 (0.41-1.08) | 0.10 |
| CCI =2 | 30/55 (54.5) | 63/108 (58.3) | 0.86 (0.45-1.65) | 0.64 | 0.98 (0.49-1.94) | 0.95 |
| CCI ≥3 | 69/136 (50.7) | 110/224 (49.1) | 1.07 (0.70-1.63) | 0.76 | 1.14 (0.73-1.79) | 0.56 |
| CVD | 114/226 (50.4) | 205/395 (51.9) | 0.94 (0.68-1.31) | 0.73 | 0.95 (0.68-1.33) | 0.75 |
| DM with complication | 100/194 (51.5) | 168/324 (51.9) | 0.99 (0.69-1.41) | 0.95 | 0.99 (0.68-1.45) | 0.97 |
| Chronic lower respiratory disease | 66/102 (64.7) | 105/153 (68.6) | 0.84 (0.49-1.42) | 0.51 | 0.83 (0.48-1.44) | 0.50 |
| Renal disease | 7/17 (41.2) | 18/40 (45.0) | 0.86 (0.27-2.70) | 0.79 | 0.82 (0.25-2.69) | 0.75 |
| Cancer | 9/24 (37.5) | 21/42 (50.0) | 0.60 (0.22-1.67) | 0.33 | 0.59 (0.20-1.76) | 0.35 |
| Urinary tract infection | 51 (17.0) | 109 (19.9) | 0.83 (0.57-1.19) | 0.31 | 0.81 (0.56-1.19) | 0.28 |
| 20 to <65 years | 21/161 (13.0) | 44/268 (16.4) | 0.76 (0.44-1.34) | 0.35 | 0.76 (0.43-1.35) | 0.35 |
| 65 to <80 years | 25/122 (20.5) | 53/247 (21.5) | 0.94 (0.55-1.61) | 0.83 | 0.90 (0.51-1.57) | 0.70 |
| ≥80 years | 5/17 (29.4) | 12/34 (35.3) | 0.76 (0.22-2.69) | 0.67 | 0.88 (0.24-3.22) | 0.85 |
| Male | 17/155 (11.0) | 49/307 (16.0) | 0.65 (0.36-1.17) | 0.15 | 0.64 (0.35-1.17) | 0.15 |
| Female | 34/145 (23.4) | 60/242 (24.8) | 0.93 (0.57-1.51) | 0.77 | 0.92 (0.56-1.51) | 0.73 |
| CCI ≤1 | 12/109 (11.0) | 25/217 (11.5) | 0.95 (0.46-1.97) | 0.89 | 1.01 (0.47-2.15) | 0.98 |
| CCI =2 | 7/55 (12.7) | 22/108 (20.4) | 0.57 (0.23-1.43) | 0.23 | 0.54 (0.21-1.40) | 0.21 |
| CCI ≥3 | 32/136 (23.5) | 62/224 (27.7) | 0.80 (0.49-1.32) | 0.38 | 0.75 (0.45-1.26) | 0.28 |
| CVD | 41/226 (18.1) | 93/395 (23.5) | 0.72 (0.48-1.08) | 0.12 | 0.70 (0.46-1.07) | 0.10 |
| DM with complications | 45/194 (23.2) | 77/324 (23.8) | 0.97 (0.64-1.47) | 0.88 | 0.89 (0.57-1.39) | 0.61 |
| Chronic lower respiratory disease | 22/102 (21.6) | 43/153 (28.1) | 0.70 (0.39-1.27) | 0.24 | 0.66 (0.36-1.21) | 0.17 |
| Renal disease | 6/17 (35.3) | 17/40 (42.5) | 0.74 (0.23-2.39) | 0.61 | 0.52 (0.15-1.87) | 0.32 |
| Pregnancy | 1/1 (100.0) | 0/5 (0.0) | - | - | - | - |
| Cancer | 5/24 (20.8) | 9/42 (21.4) | 0.96 (0.28-3.30) | 0.95 | 0.95 (0.26-3.52) | 0.94 |
| Genital infections | 36 (12.0) | 38 (6.9) | **1.83 (1.14-2.96)** | **0.01** | **1.76 (1.07-2.90)** | **0.03** |
| 20 to <65 years | 22/161 (13.7) | 28/268 (10.4) | 1.36 (0.75-2.46) | 0.31 | 1.32 (0.71-2.44) | 0.38 |
| 65 to <80 years | 11/122 (9.0) | 10/247 (4.0) | 2.35 (0.97-5.69) | 0.05 | 2.19 (0.86-5.55) | 0.10 |
| ≥80 years | 3/17 (17.6) | 0/34 (0.0) | - | - | - | - |
| Male | 1/155 (0.6) | 1/307 (0.3) | - | - | - | - |
| Female | 35/145 (24.1) | 37/242 (15.3) | **1.76 (1.05-2.96)** | **0.03** | 1.66 (0.98-2.84) | 0.06 |
| CCI ≤1 | 14/109 (12.8) | 17/217 (7.8) | 1.73 (0.82-3.66) | 0.15 | 1.57 (0.72-3.43) | 0.26 |
| CCI =2 | 11/55 (20.0) | 8/108 (7.4) | **3.13 (1.18-8.30)** | **0.02** | 2.61 (0.95-7.14) | 0.06 |
| CCI ≥3 | 11/136 (8.1) | 13/224 (5.8) | 1.43 (0.62-3.29) | 0.40 | 1.62 (0.67-3.97) | 0.29 |
| CVD | 25/226 (11.1) | 18/395 (4.6) | **2.61 (1.39-4.89)** | **0.002** | **2.29 (1.19-4.37)** | **0.01** |
| DM with complications | 15/194 (7.7) | 23/324 (7.1) | 1.10 (0.56-2.16) | 0.79 | 1.22 (0.59-2.53) | 0.59 |
| Chronic lower respiratory disease | 17/102 (16.7) | 12/153 (7.8) | **2.35 (1.07-5.16)** | **0.03** | 2.11 (0.93-4.76) | 0.07 |
| Renal disease | 1/17 (5.9) | 1/40 (2.5) | - | - | - | - |
| Pregnancy | 0/1 (0.0) | 4/5 (80.0) | - | - | - | - |
| Cancer | 3/24 (12.5) | 2/42 (4.8) | - | - | - | - |

Notes: Risk analyses were adjusted for comedication patterns of sulfonylurea, thiazolidinedione, and glucagon-like peptide-1 analog. p-values were calculated with Chi-square test (Fisher’s exact test) for categorial variables. Statistically significant p-values are highlighted in bold.

Abbreviations: PS, propensity score; SGLT2i, sodium glucose cotransporter-2 inhibitor; T2DM, type 2 diabetes mellitus; CCI, Charlson comorbidity index; CVD, cardiovascular disease; DM, diabetes mellitus.

Table S4. Index dates: DPP-4 Inhibitor + Insulin vs Metformin + Insulin

| Index Date (2019) | DPP4i+Insulin | Metformin+Insulin |
| --- | --- | --- |
| Jan | 1020 | 516 |
| Feb | 279 | 141 |
| Mar | 112 | 61 |
| Apr | 40 | 20 |
| May | 26 | 9 |
| Jun | 21 | 2 |
| Total | 1498 | 749 |

Table S5. Index dates: SGLT-2 Inhibitor + Insulin vs Metformin + Insulin

| Index Date (2019) | SGLT2i+Insulin | Metformin+Insulin |
| --- | --- | --- |
| Jan | 208 | 373 |
| Feb | 56 | 103 |
| Mar | 25 | 48 |
| Apr | 8 | 15 |
| May | 3 | 8 |
| Jun |  | 2 |
| Total | 300 | 549 |

< Group 1: Metformin + Insulin vs. Group 2: DPP-4 Inhibitor + Insulin >


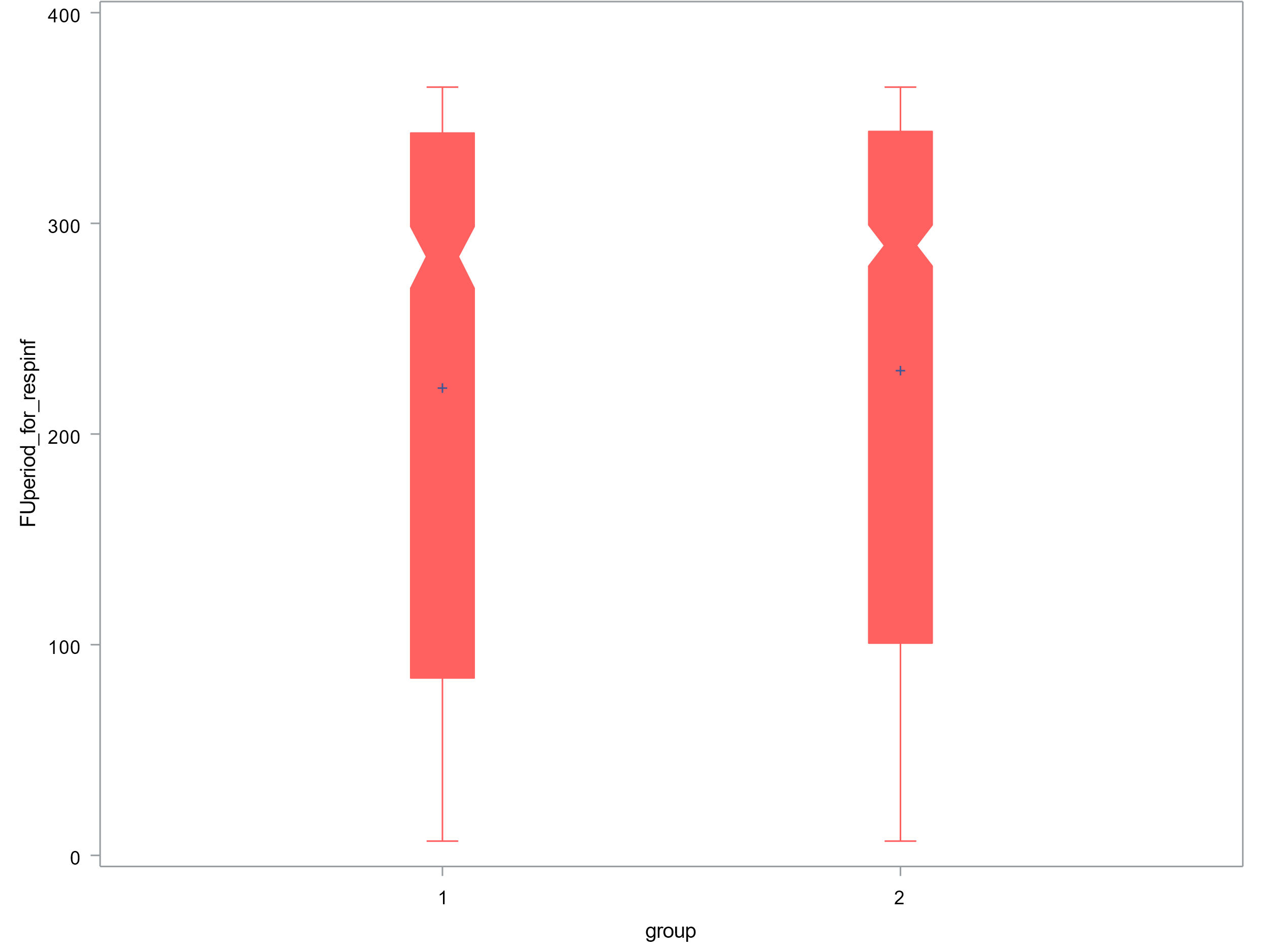


Figure S1. Follow-Up Periods for Respiratory Infection:


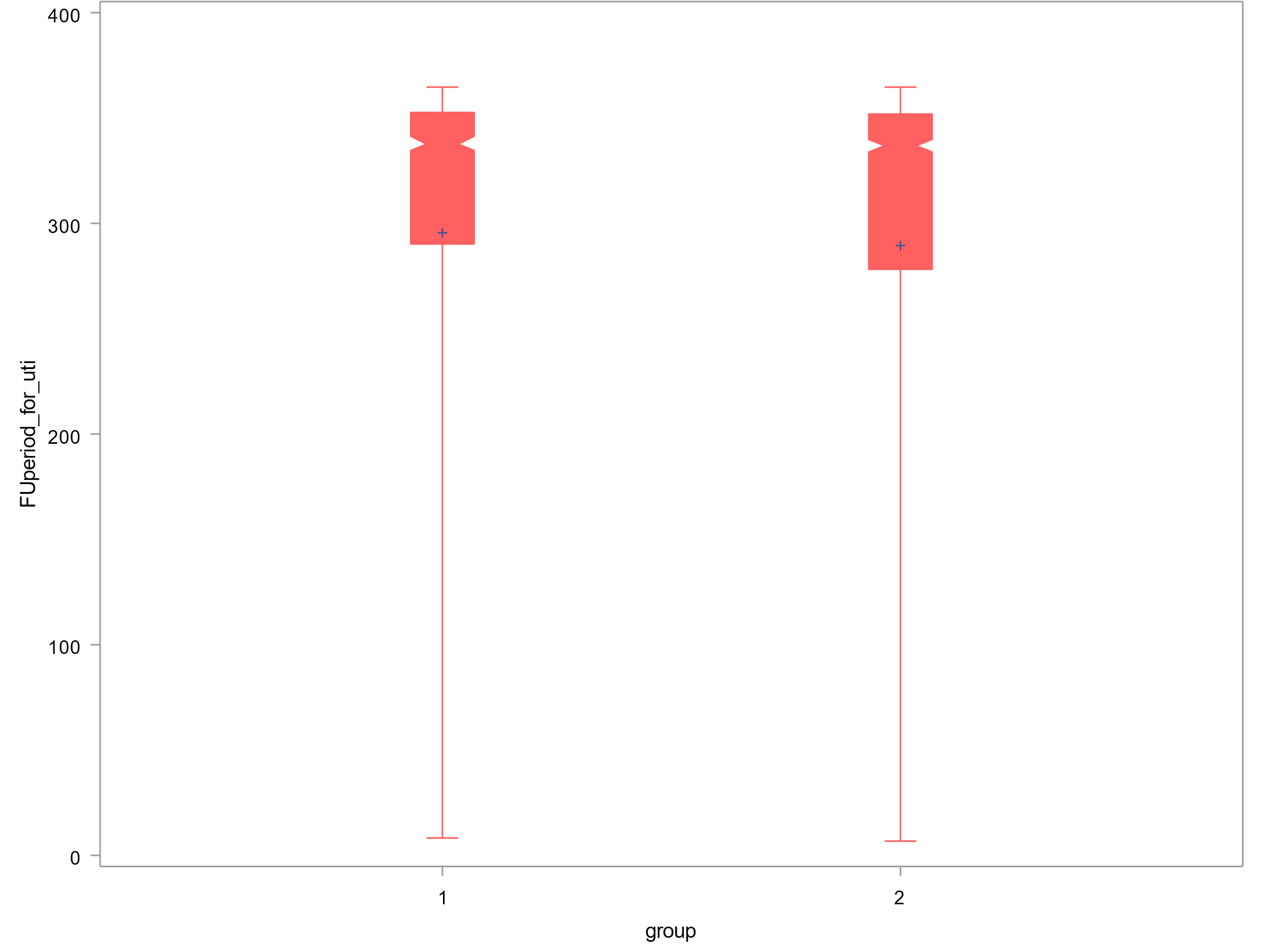


Figure S2. Follow-Up Periods for Urinary Tract Infection


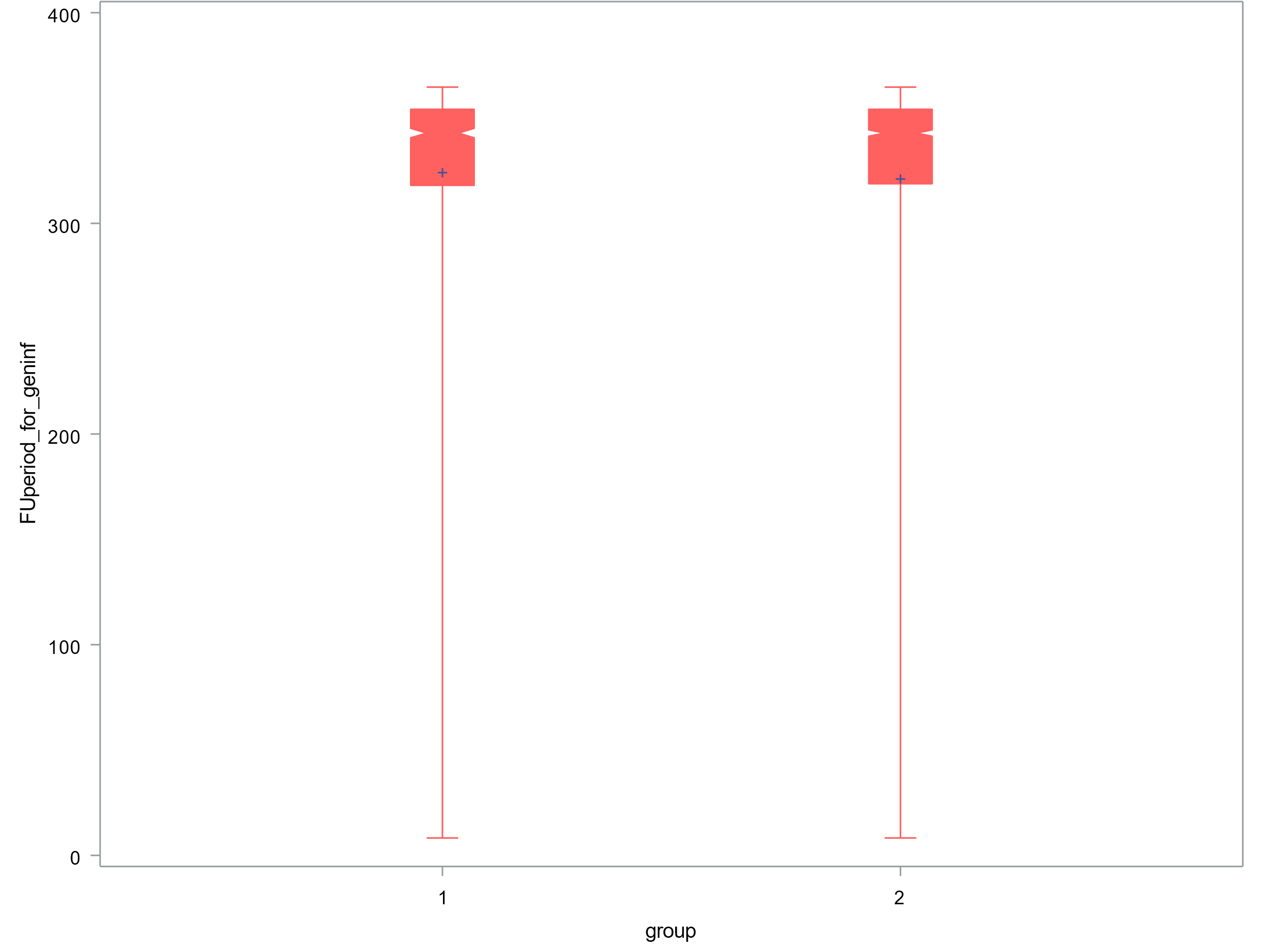


Figure S3. Follow-Up Periods for Genital Infection

< Group 1: Metformin + Insulin vs. Group 3: SGLT-2 Inhibitor + Insulin >


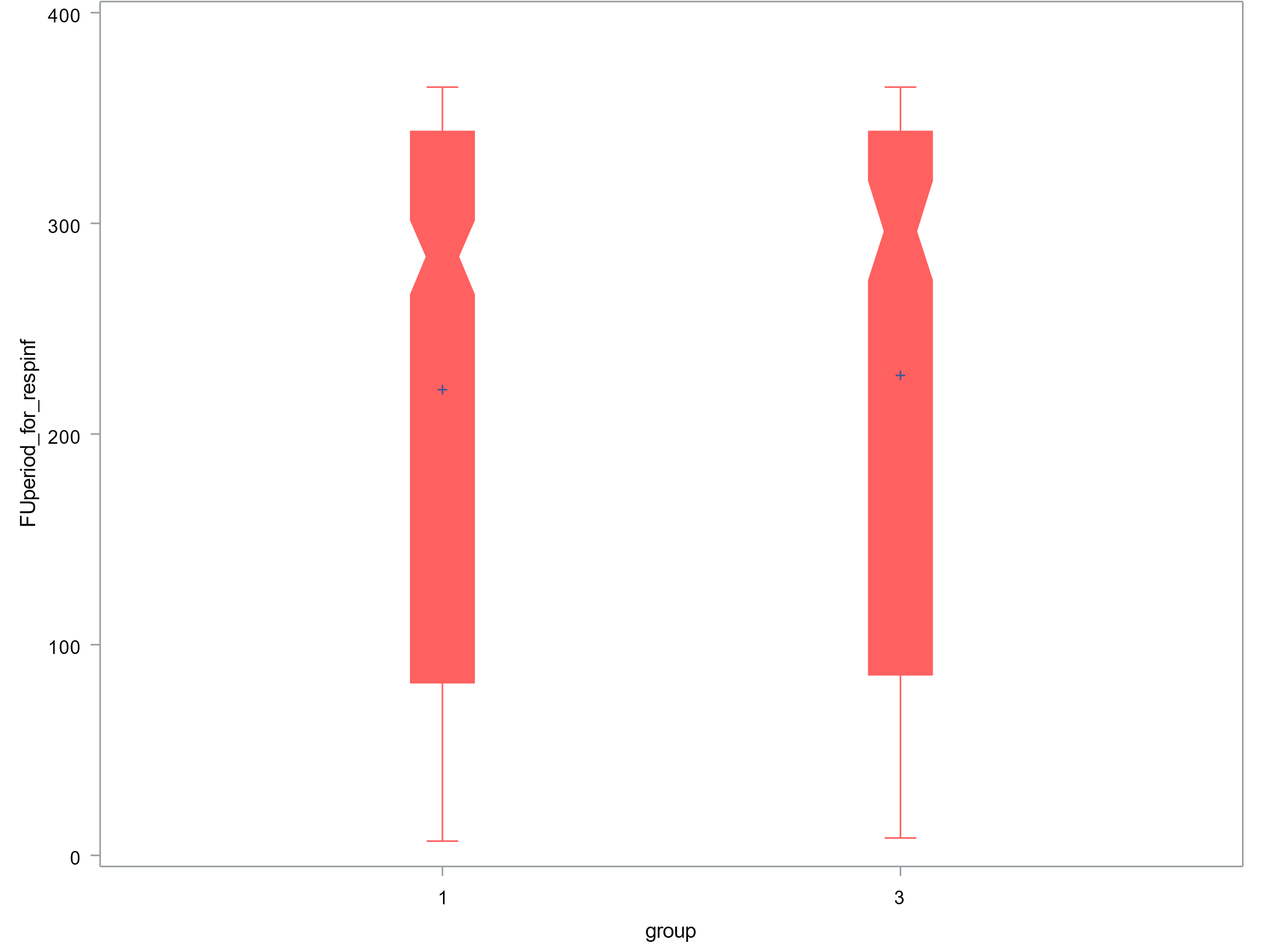


Figure S4. Follow-Up Periods for Repiratry Infection


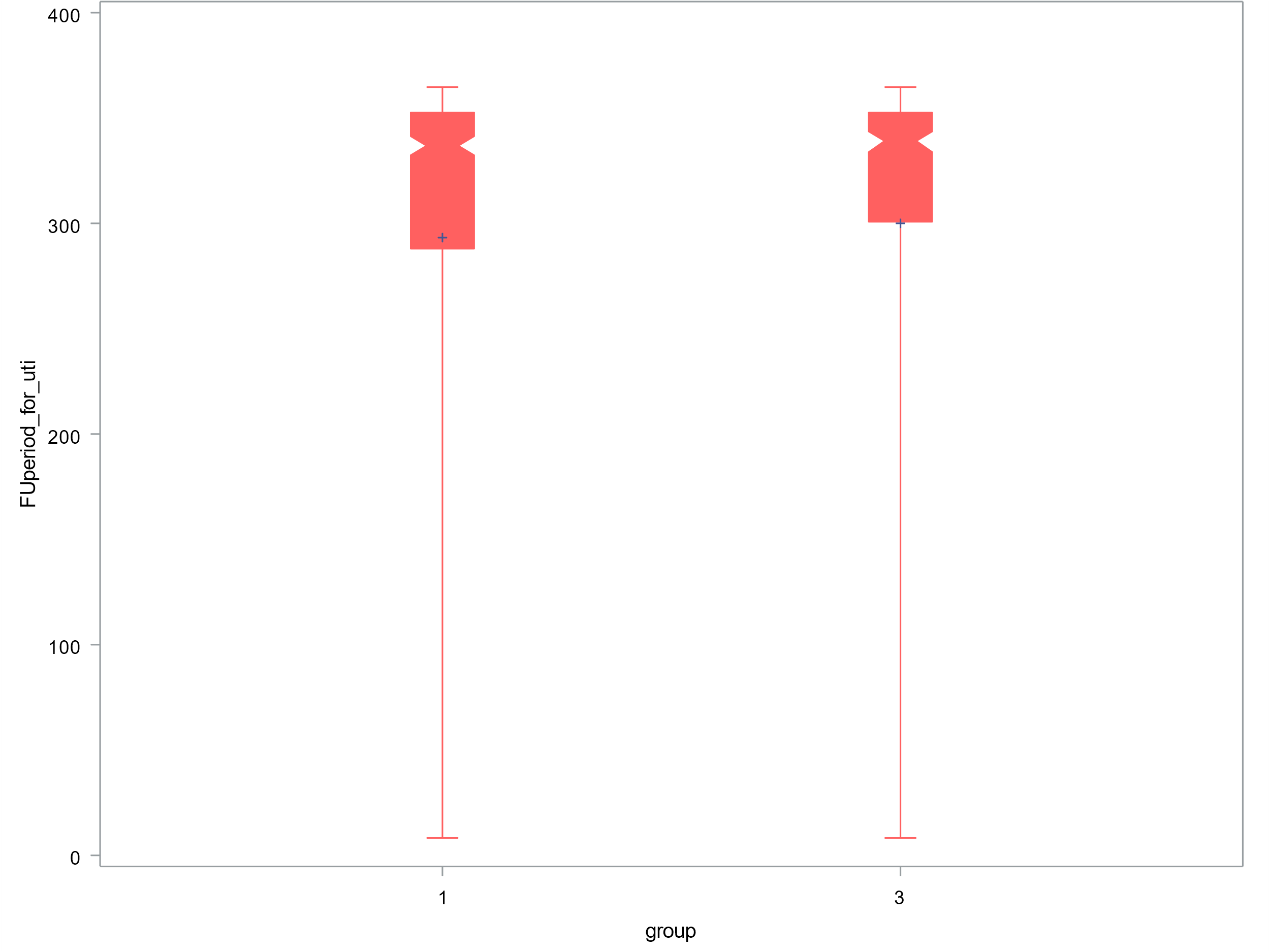


Figure S5. Follow-Up Periods for Urinary Tract Infection


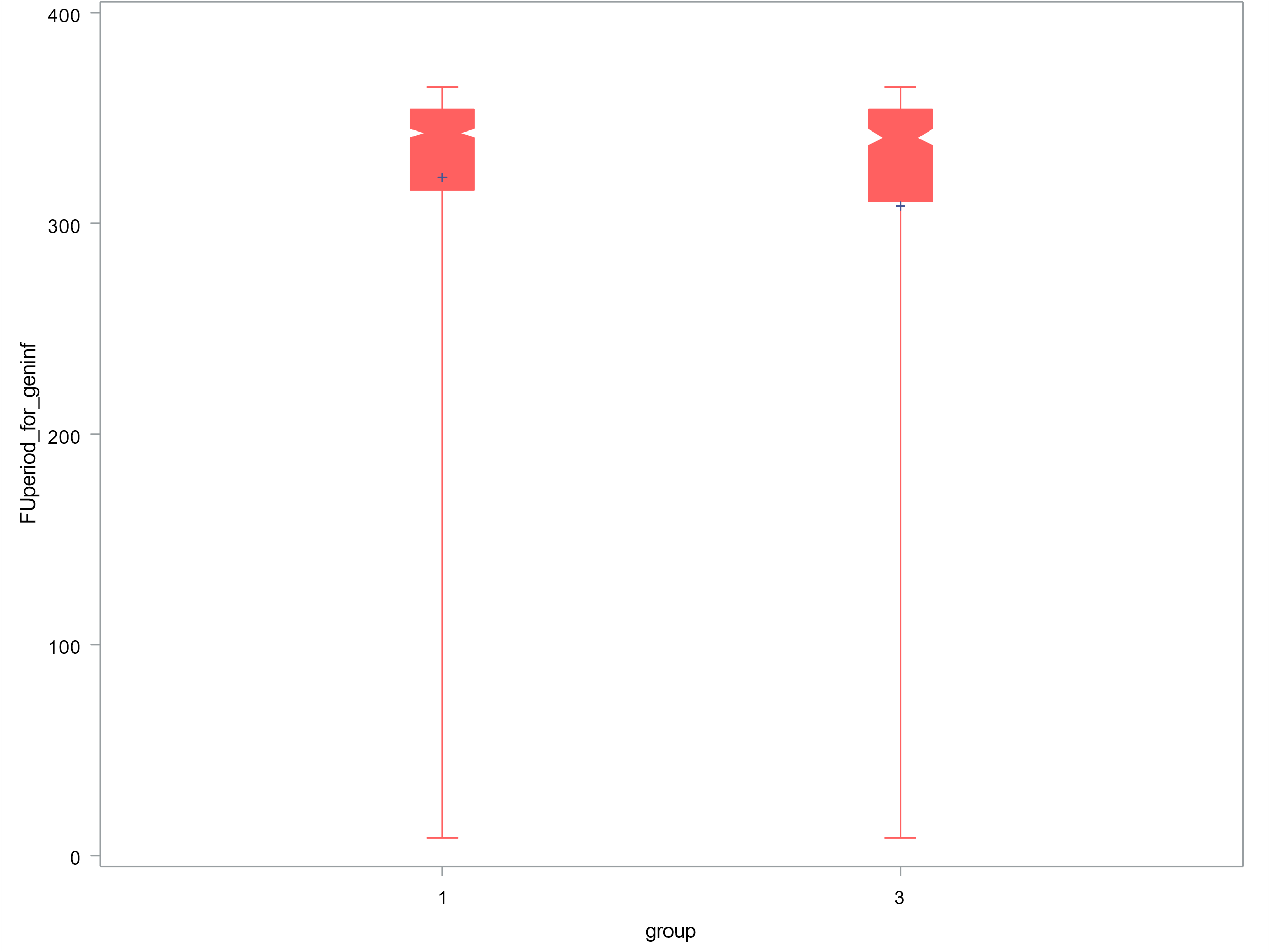


Figure S6. Follow-Up Periods for Genital Infection
